# *Saccharomyces cerevisiae* mitochondria are required for optimal attractiveness to *Drosophila melanogaster*

**DOI:** 10.1371/journal.pone.0113899

**Published:** 2014-12-02

**Authors:** Kelly M. Schiabor, Allison S. Quan, Michael B. Eisen

**Affiliations:** 1 Department of Molecular and Cell Biology, University of California, Berkeley, California 94720, United States of America; 2 Department of Integrative Biology, University of California, Berkeley, California 94720, United States of America; 3 QB3 Institute, University of California, Berkeley, California 94720, United States of America; 4 Howard Hughes Medical Institute, University of California, Berkeley, California 94720, United States of America; Auburn University, UNITED STATES

## Abstract

While screening a large collection of wild and laboratory yeast strains for their ability to attract *Drosophila melanogaster* adults, we noticed a large difference in fly preference for two nearly isogenic strains of *Saccharomyces cerevisiae*, BY4741 and BY4742. Using standard genetic analyses, we tracked the preference difference to the lack of mitochondria in the BY4742 strain used in the initial experiment. We used gas chromatography coupled with mass spectroscopy to examine the volatile compounds produced by BY4741 and the mitochondria-deficient BY4742, and found that they differed significantly. We observed that several ethyl esters are present at much higher levels in strains with mitochondria, even in fermentative conditions. We found that nitrogen levels in the substrate affect the production of these compounds, and that they are produced at the highest level by strains with mitochondria when fermenting natural fruit substrates. Collectively these observations demonstrate that core metabolic processes mediate the interaction between yeasts and insect vectors, and highlight the importance mitochondrial functions in yeast ecology.

## Introduction

Since the early 20th century, the vinegar fly *Drosophila melanogaster* and the brewer’s yeast *Saccharomyces cerevisiae*, have been the preeminent model species of molecular biology. Although generally studied by separate sets of researchers in the lab, these species are found together in nature on fermenting fruit substrates—an interaction that has received surprisingly little attention.

Their co-occurrence is no coincidence: each species benefits from the presence of the other. Ethanol produced by *S. cerevisiae* protects *D. melanogaster* larvae from predators [1], and *D. melanogaster* larvae preferentially feed on *S. cerevisiae*, which provides a complete nutritional source for larval development [2,3]. The non-motile *S. cerevisiae*, in turn, rely on *D. melanogaster* and other insects to move to fresh, nutrient-rich substrates [4], and the burrowing activities of *D. melanogaster* larvae expose nutritional sources to the yeast that would have been otherwise inaccessible [5].

Given the benefits that each species gains from this interaction, it is not surprising that both yeast and fly appear to have evolved molecular mechanisms to actively maintain their co-localization.

Anyone who keeps fruits in their kitchen will know that *D. melanogaster* are attracted to fermenting fruit. This effect is mediated almost entirely by volatile molecules produced by yeasts, and not the fruity substrate [2], although the substrate can have a significant effect on what compounds are produced[6–12]. Numerous volatile compounds produced by *S. cerevisiae* activate specific odorant receptor neurons in the *D. melanogaster* antennae, which are relayed to the odor representation center of the fly brain, the antennal lobe [13,14], and elicit specific attractive and repulsive responses [2] [15–22].

The response to individual compounds is complex, and includes various types of attraction and avoidance behaviors. For example, a small pulse of ethyl butyrate is sufficient to keep a fly flying towards the source of the odorant for twenty minutes without additional application of the odor, but this tracking is much less pronounced for acetic acid, another attractive yeast-produced volatile [17]. Mixtures of yeast-produced volatile compounds, more akin to what flies would experience in nature, produce more complex, context-dependent responses [16].

Different yeast species produce varying volatile bouquets, even when grown on identical substrates [6,8,12,23–26]. This variance in volatile bouquets is observed across *S. cerevisiae* strains as well [24]. Further, fly species, and also different *D. melanogaster* strains, show varying preferences for yeast species and strains [27–29].

We became interested in the possibility that the large repertoire of genetic tools available for *D. melanogaster* and *S. cerevisiae* would allow us to exploit these different types of variation to better understand the molecular details of this interaction. As a first step, we examined the response of a wild-caught, un-starved *D. melanogaster* strain to different *S. cerevisiae* strains grown under identical conditions.

## Results

### Two nearly isogeneic lab strains vary in their attractiveness to *D. melanogaster*

**Figure 1:**
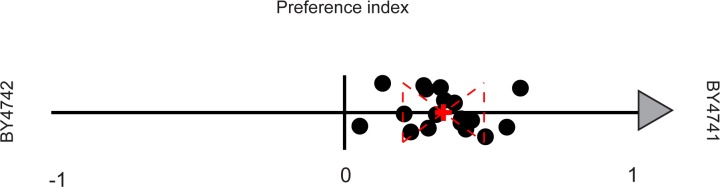
*D. melanogaster* are preferentially attracted to BY4741 over nearly isogenic BY4742. We compared *D. melanogaster* preference for different yeast strains using an open-flight preference assay. Each black dot represents the results of a single open-flight preference trial between lab stocks of BY4741 and BY4742. The preference index (PI) for each trial is defined as: PI = (A−B)/(A+B/2), where A = total flies caught in BY4741 baits and B = total flies caught in BY4742 baits. This value represents the direction of the preference (positive for A, negative for B) as well as the normalized degree of preference by the percentage over the expected. Red dashed lines represent the two-tailed standard deviation and the red “+” represents the mean of all trials.

We used a simple preference assay (see Methods) to screen a large panel of *S. cerevisiae* strains, which were collected from diverse environments with varied histories of laboratory use and propagation, for their attractiveness to a recently wild-caught, un-starved *D. melanogaster* line (Raleigh 437). We found considerable variation in the attractiveness across the strains (corroborating the results of [24]). As we were ultimately interested in exploring the genetic basis (in yeast) for this varied *D. melanogaster* attractiveness, we also included the two nearly isogeneic haploid *S. cerevisiae* strains, BY4741 and BY4742, which had been used to generate the systematic yeast deletion collection [30]. We were surprised to find that traps baited with BY4741, the MAT a parent, consistently attracted more flies when directly competing against traps baited with BY4742, the MAT*α* parent (Fig. 1). The remainder of this paper is focused on understanding the origins of this preference difference.

### Attraction difference between BY4741 and BY4742 is associated with functional mitochondria

**Figure 2:**
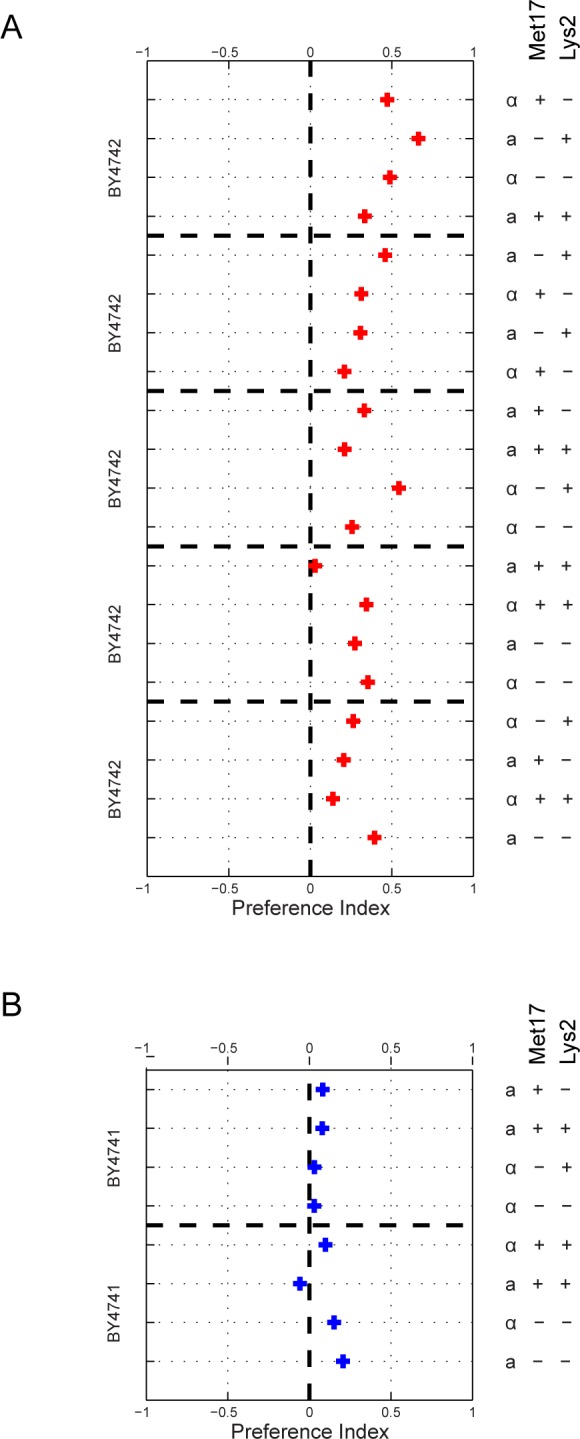
Genetic analysis of *D. melanogaster* preference for BY4741 over BY4742 suggests mitochondrial inheritance. A) To test if behavioral preference is associated with genotype, BY4741 and BY4742 were crossed and sporulated and the resulting haploid segregants genotyped at the MAT, MET15, and LYS2 loci. Segregants from five tetrads, collectively covering spores of every genotype, were tested against BY4742 in our behavioral assay. The red “+” represents the mean preference index of three trials. Tetrad spores of all genotypes phenocopy the BY4741 parent, suggesting a mitochondrial inheritance pattern. B) Two tetrads were tested against both BY4741 (blue “+”s), with the “+” representing the mean preference index of five behavioral trials. We found no preference between each tetrad spore and BY4741, all of which possess functional mitochondria. As shown in (A), all spores were preferred over BY4742, which does not possess functional mitochondria. T-tests were preformed for each genotype against both BY4742 and BY4741 (Table 1).

Outside of the mating type locus, these strains ostensibly differed at two metabolic marker loci: MET17 (deleted in BY4741) and LEU2 (deleted in BY4742). Given the known relationship between amino acid metabolism and volatile compound production [6,9,10,31–35], we suspected that the difference in behavioral response to these two strains could be traced to their respective auxotrophies. We therefore crossed BY4741 and BY4742 and dissected several tetrads to obtain haploid strains with all eight possible genotypes at the three variable loci (MAT, LEU2 and MET15). However, when we tested these strains using our choice assay against BY4742, we found that strains representing all eight genotypes were strongly preferred to BY4742, including the MAT*α*; MET15; leu2Δ strain, which has the identical three-locus genotype as BY4742 (Fig. 2A). We tested a subset of these strains against BY4741, and found that the flies preferred each strain equally (Fig. 2B). Statistics were performed by pooling trial results for each genotype. All genotypes are significantly preferred over BY4742, while no significant preference difference was detected for haploid strains tested against BY4741 (Table 1).

**Table 1:** Statistics to accompany Fig. 2.

**Sample 1**	**Sample 2**	**Mean 1**	**Std 1**	**Mean 2**	**Std 2**	**Trials**	**Ttest**	**Pval**
MET;LYS;MATA	BY4742	68	27	35	19	9	1	4.67E-02
MET;LYS;MATAL	BY4742	66	20	38	15	12	1	1.53E-02
met;LYS;MATA	BY4742	83	13	30	12	9	1	7.42E-05
met;LYS;MATAL	BY4742	75	16	32	12	6	1	5.37E-03
MET;lys;MATA	BY4742	68	17	36	12	12	1	1.31E-03
MET;lys;MATAL	BY4742	78	13	39	10	9	1	6.80E-04
met;lys;MATA	BY4742	74	16	34	12	6	1	9.39E-03
met;lys;MATAL	BY4742	68	24	32	15	9	1	9.46E-03
MET;LYS;MATA	BY4741	57	18	57	18	9	0	9.78E-01
MET;LYS;MATAL	BY4741	63	15	51	14	5	0	4.19E-01
met;LYS;MATAL	BY4741	59	12	54	6	4	0	6.68E-01
MET;lys;MATA	BY4741	60	10	51	11	4	0	4.66E-01
met;lys;MATA	BY4741	65	19	44	23	5	0	3.17E-01
met;lys;MATAL	BY4741	63	16	52	15	9	0	2.95E-01

This non-autosomal inheritance pattern immediately suggested mitochondrial linkage. To confirm a mitochondrial difference, we sequenced both parental strains as well as an independent BY4742 isolate and mapped the sequence reads back to the *S. cerevisiae* reference genome. Sequence reads from both BY4741 and the independent BY4742 isolate mapped across the entire mitochondrial genome, while virtually no reads from the BY4742 parent strain mapped to the mitochondrial genome (S1 Table). Additionally, we tested both parental strains and all progeny for growth on glycerol, a non-fermentable carbon source, and found that all of the strains grew normally, except for BY4742 (Data not shown). Together these results confirm that our BY4742 parent lacks a mitochondrial genome and subsequently cannot respire. We will refer to this parental strain hereon as BY4742p, for petite.

**Figure 3:**
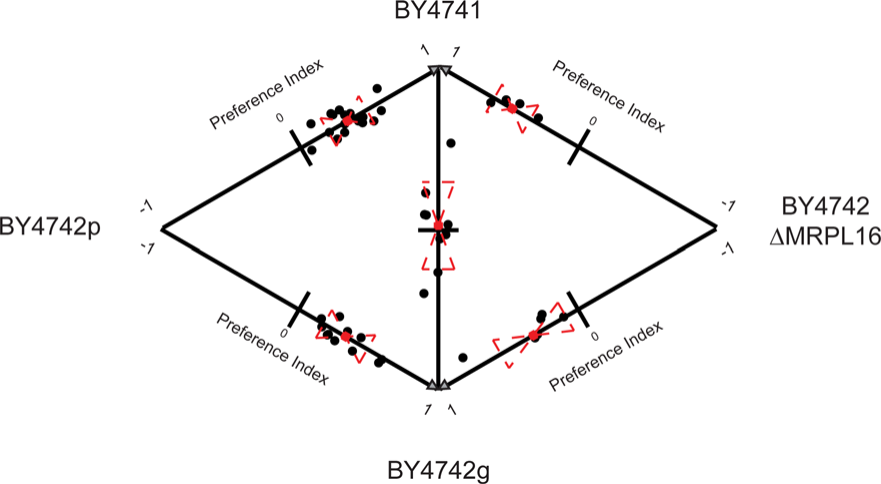
Mitochondrial status of *S. cerevisiae* affects *D. melanogaster* preference. Pairwise behavioral results between BY4741 and various BY4742 strains, each with a different mitochondrial status confirm role of mitochondria in *D. melanogaster* preference. BY4742p is the original isolate, BY4742g is an independent BY4742 strain with functional mitochondria, and BY4742 ΔMRPL16 is a strain from the deletion collection known to genetically disrupt mitochondrial function. Each black dot represents the preference index for each trial (see Fig. 1). Red dashed lines represent the two-tailed standard deviation and the red “+” represents the mean of all trials. T-tests were performed for every pairwise trial (Table 2).

To corroborate that the absence of a functional mitochondria reduces the attractiveness of yeast strains to *D. melanogaster*, we tested the independent BY4742 isolate (BY4742g, for grande), with a functional mitochondria (*MATα;his3;leu2;lys2;ura3*), as well the MRPL16Δ strain (*MATα;his3;leu2;lys2;ura3;mrpl16*) from the same deletion collection. MRPL16 encodes for the large subunit of the mitochondrial ribosomal protein, the deletion of which results in a respiration deficiency. We found that BY4742g was equally as attractive as BY4741, while the MRPL16Δ strain was less attractive than BY4741. Following the trend found with BY4741, we found that BY4742g was also more attractive than both BY4742p and the MRPL16Δ strain (Fig. 3). Finally, the BY4742p and MRPL16Δ strains, both with respiration deficiencies, were equally preferred (Table 2). This series of results further confirms that *D. melanogaster* are more attracted to S. *cerevisiae* strains possessing functional mitochondria.

**Table 2:** Statistics to accompany Fig. 3.

**Sample 1**	**Sample 2**	**Mean 1**	**Std 1**	**Mean 2**	**Std 2**	**Trials**	**Ttest**	**Pval**
BY4741	BY4742g	57	17	53	17	9	0	7.73E-01
BY4741	BY4742p	76	17	37	11	18	1	1.19E-07
BY4742g	BY4742p	71	10	35	9	11	1	3.61E-05
BY4742g	ΔMRPL16	77	4	41	1	2	1	4.35E-02
BY4741	ΔMRPL16	89	6	31	9	4	1	3.94E-03
BY4742p	ΔMRPL16	61	18	60	14	2	0	9.86E-01

### BY4741 and BY4742p strains produce different volatile bouquets, influenced by their mitochondrial status

**Figure 4:**
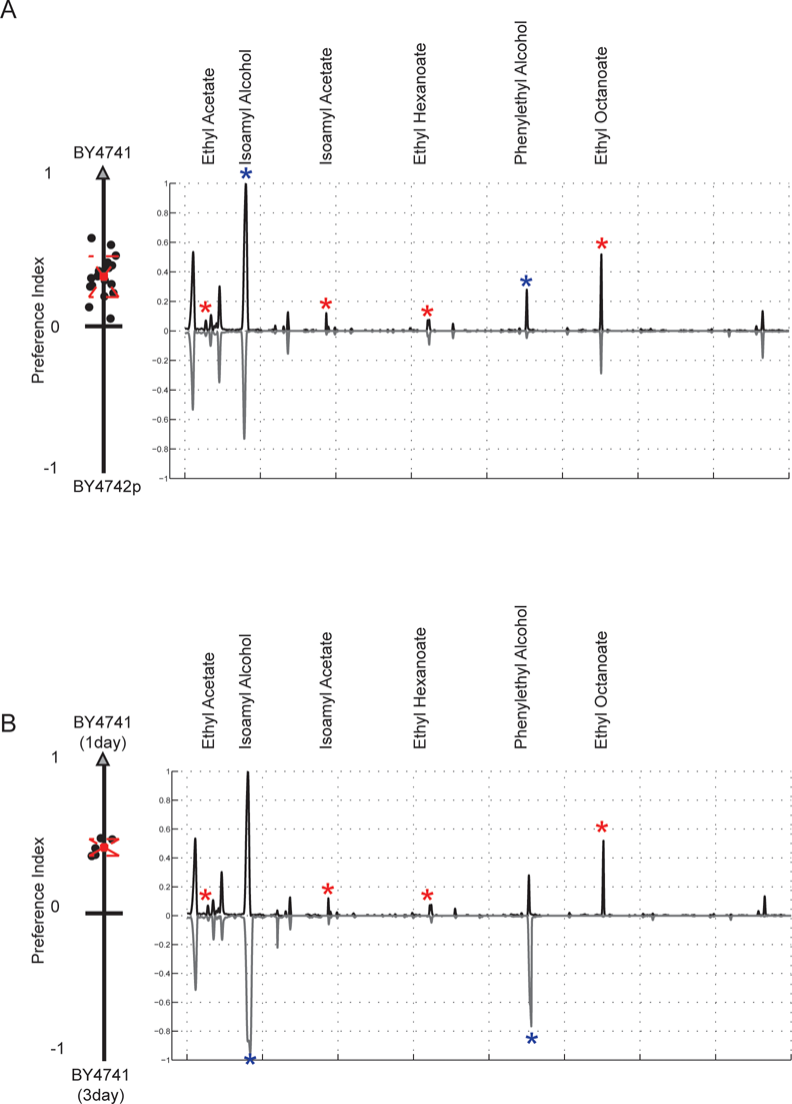
GC-MS identifies volatile compounds that likely underlie *D. melanogaster* preference for *S. cerevisiae* strains with functional mitochondria. A) To evaluate the effect of mitochondrial status on volatile compound production, we performed GC-MS on one day old BY4741 and BY4742p cultures. The average total ion chromatograms (TIC) of 5 biological replicates for BY4741 is plotted on the positive y-axis, while the average TIC for BY4742p is plotted on the negative y-axis. The preference data from Fig. 1 for these two strains is shown to the left of the panel. Peaks marked with stars represent compounds at higher abundance in BY4741 and represent potential attractants. Compound names are given for all starred peaks. Peak identification statistics are reported in Table 3 and comparison to synthetic standards in S6 Fig. B) To evaluate if the differences were due to the respiration defect in BY4742p, we used GC-MS to compare the volatile profiles of one-day-old BY4741 cultures and respiration-dominated, three day old BY4741 cultures; *D. melanogaster* exhibits a strong preference for the one-day-old cultures. The one day BY4741 TIC is plotted on the positive y-axis, and the three-day-old BY4741 TIC on the negative y-axis. One day BY4741 cultures produce higher levels of a subset of the attractants (red stars), while the others grow in the three-day cultures (blue stars). T-tests were preformed for all behavioral trials (Table 4).

In the choice assay used here, *D. melanogaster* selected between different yeast strains based primarily on how they smell. Thus, the absence of functional mitochondria must be affecting the volatile compounds produced by BY4742p. We used Gas Chromatography coupled to Mass Spectroscopy (GC-MS) to characterize and quantify the volatile compounds associated with both BY4741 and BY4742p grown on YPD plates, and, as expected, found significant differences (Fig. 4A). Specifically, six compounds were consistently found at higher levels in BY4741 than BY4742p: ethyl acetate, isoamyl alcohol, isoamyl acetate, ethyl hexanoate, phenylethyl alcohol, and ethyl octanoate (Table 3). A similar result was obtained when comparing the progeny of the BY4741 × BY4742p cross (S1 Fig.). These compounds have all previously been identified as attractants for *D. melanogaster* [14,21,24].

**Table 3:** Identified chemical attractants.

**Compound**	**CAS#**	Retention Time (min)*	NIST*	MW*	Quality (% Match)*
Ethyl Acetate †	141-78-6	2.6	2019	88	86
Isoamyl Alcohol	123-51-3	4	2124	88	90
Isoamyl Acetate †	123-92-2	7.5	13474	130	90
Ethyl Hexanote †	123-66-0	11.7	20902	144	98
Phenylethyl Alcohol	60-12-8	15.7	9921	122	91
Ethyl Octanoate †	106-32-1	18.8	39512	172	90

* Retention time, NIST, Molecular Weight (MW), and Quality are based on BY4741 chromatogram (061014_02.CDF)

† Compounds verified with synthetic standards. See GC-MS methods and S6 Fig.

**Table 4:** Statistics to accompany Fig. 4.

**Sample 1**	**Sample 2**	**Mean 1**	**Std 1**	**Mean 2**	**Std 2**	**Trials**	**Ttest**	**Pval**
BY4741 1 day	BY4742p 1 day	76	17	37	11	18	1	1.19E-07
BY4741 1day	BY4741 3 day	81	6	31	4	5	1	7.21E-05

To determine the extent to which these volatile profile changes were directly related to mitochondrial engagement, we examined the volatile profiles of BY4741 and BY4742p in liquid YPD cultures under both anaerobic and aerobic conditions. As expected, the anaerobic and aerobic cultures of BY4742p, which cannot respire, produced nearly identical chromatograms (S2 Fig.). In contrast, the anaerobic and aerobic BY4741 cultures were quite different (S2 Fig.). The anaerobic BY4741 culture resembled the BY4742p culture, and the aerobic BY4741 culture resembled BY4741 grown on plates. In particular, the aerobic BY4741 culture showed a marked increase, relative to the BY4741 anaerobic culture, in the six attractive compounds that differed between BY4741 and BY4742p grown on plates.

The effect of mitochondria on the volatile profile of a strain is an unexpected result. *S. cerevisiae* exhibit a phenomenon known as the Crabtree effect, in which they preferentially ferment glucose to ethanol, even in the presence of oxygen [36,37]. Thus, we would not expect the absence of a functional mitochondria to have a significant effect until available glucose has been depleted and the yeast are forced to respire. However, our data clearly indicate that the mitochondria are having a significant phenotypic effect during both fermentation and respiration.

### Respiration alone does not explain *D melanogaster* preference for BY4741 over BY4742p

One possible explanation is that there is a low level of respiration in cultures that are primarily fermenting. If respiration alone is driving attraction, then we would expect older cultures, which have depleted their glucose levels and will have higher rates of respiration, to be more attractive. However, when we compared cultures of BY4741 at different times following inoculation, we found exactly the opposite: younger cultures of BY4741 are more attractive than older cultures, suggesting that attractiveness is not entirely respiration dependent (Fig. 4B and Table 4).

When we compared the volatile compounds produced by the older and younger cultures using GC-MS we found that two of the potential attractants (isoamyl alcohol, phenylethyl alcohol) continued to grow in concentration in the older, respiring cultures, while the rest (isoamyl acetate, and the ethyl esters) disappeared (Fig. 4B). These results suggest that individual metabolites vary in levels of attractiveness. We classified the esters: ethyl acetate, isoamyl acetate, ethyl hexanoate, and ethyl octanoate, which are highest in the younger plates, as primary attractants. We classified isoamyl alcohol and phenylethyl alcohol as secondary attractants, as our results suggest these compounds are not as attractive as the esters. Additionally, knocking out EEB1, a gene known to affect ethyl ester production, in a BY4741 background decreases the production of our identified attractants and subsequently affects fly preference (S3 Fig.) [38,39].

Overall, these observations suggest that high levels of esters produced by BY4741 during our early time point are primarily driving attraction, and that they are not strictly associated with respiration. Instead, they appear to arise from a separate mitochondria-linked process.

### Nitrogen source affects both the production of identified attractants and fly preference

If respiration is not directly driving the production of these attractants, what is? Although it is not widely appreciated, the mitochondria is also required for nitrogen metabolism [40]. For example, both proline catabolism and branched chain amino acid anabolism have biochemical steps that occur within the mitochondria [41,42]. We therefore hypothesized that varying nitrogen sources might impact volatile compound production.

**Figure 5:**
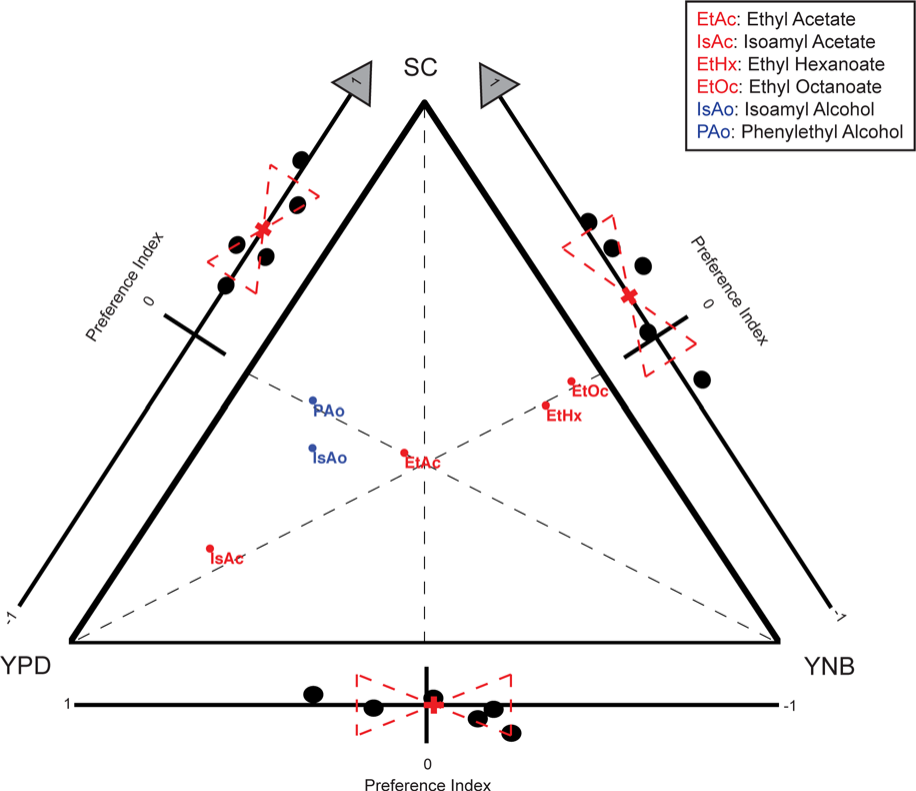
Nitrogen source affects levels of identified attractants and fly behavioral preference in a prototrophic *S. cerevisiae* strain. We performed pairwise preference assays and GC-MS on a prototrophic strain of *S. cerevisiae* (T73) grown on yeast extract, peptone, dextrose media (YPD), yeast nitrogen base media (YNB) and synthetic complete media (SC) which vary in types and levels of nitrogen. The pairwise *D. melanogaster* behavioral results have been added to each side of the triangle representing the three pairwise comparisons. GC-MS data is plotted using triangular Barycentric coordinates (the point for a given compound is placed at the center of mass if the vertices are assigned a mass corresponding to the GC-MS signal for that compound in the corresponding sample). The variation in each attractant for every pairwise comparison can be read by looking at how the compounds segregate across the midpoint of the relevant side (drawn on figure as dashed line for emphasis). Primary attractants are plotted in red and secondary attractants are plotted in blue. Attractants, in combination, track with observed behavioral preferences, but no single attractant can explain all behavioral results.

To test this we grew a prototropic, wild caught *S. cerevisiae* strain, T73, on media containing equivalent carbon source (5% glucose) and varying types and levels of nitrogen (Table 6). We collected GC-MS data for each condition and tested all pairwise comparisons between these media in our behavioral assay. We found that varying the nitrogen source had a dramatic effect on the types and levels of volatile compounds produced by the culture, including the identified attractants from our BY4741 strain (Fig. 5). We confirmed the same trends in additional wild strains isolated from various environments (S4 Fig.).

In addition, the varying levels of the six attractive compounds produced by these cultures confirmed our earlier behavior results: in pairwaise comparisons, the culture that produced higher combined levels of attractive compounds was always more attractive to *D. melanogaster* (Fig. 5 and Table 5).

**Table 5:** Statistics to accompany Fig. 5 and Fig. 6.

**Sample 1**	**Sample 2**	**Mean 1**	**Std 1**	**Mean 2**	**Std 2**	**Trials**	**Ttest**	**Pval**
T73 Grape	T73 SC	66	13	49	10	4	0	2.28E-01
T73 SC	T73 YNB	66	15	48	12	5	0	2.07E-01
T73 SC	T73 YPD	74	9	33	10	5	1	5.93E-03
T73 YPD	T73 YNB	55	12	57	13	6	0	8.14E-01

### A wild strain grown on a natural fruit substrate produces the highest levels of identified attractants

Interestingly, across all pairwise comparisons, the most attractive medium tested was the synthetic complete medium (SC), which most closely matched the nutrient composition of a fruit, the natural substrate of *S. cerevisiae*. Like fruit, SC media contained a mixed pool of amino acids as a nitrogen source and high levels of sugar (5% glucose) (Table 6). This is in stark contrast to traditional laboratory growth media, YPD, which is limited in sugar but rich in inorganic nitrogen.

**Figure 6:**
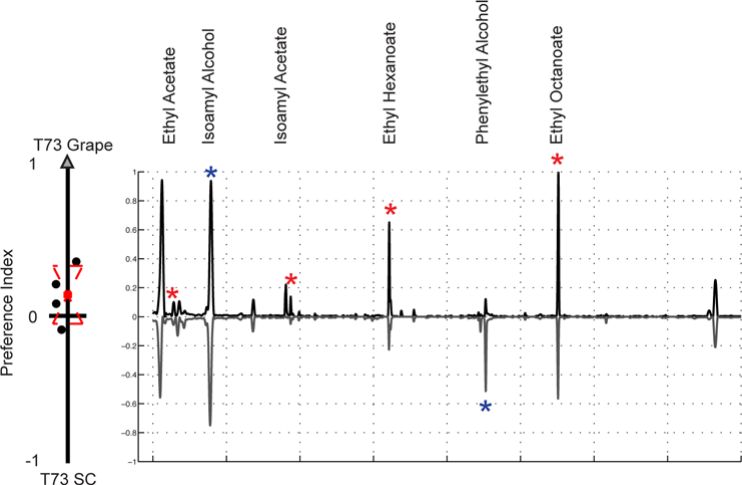
Primary attractants are detected at extremely high levels when T73 is grown on its natural substrate, grape. Behavioral results comparing the prototrophic strain T73 grown on grape media and synthetic complete media (SC) (left). Average TIC for GC-MS data of T73 grown on grape media (positive y-axis) and on synthetic complete media (SC) (negative y-axis). Both TICs were normalized together so that the maximum value across both experiments was set to one.

Based on the results of our nitrogen experiments, we collected GC-MS profiles of T73 grown on pureed grape agar. The GC-MS profiles for these cultures were dominated by our identified attractants. These cultures contained the highest level of ethyl esters and isoamyl acetate seen in this study (Fig. 6). These grape-based fermentations were extremely attractive to *D. melanogaster* and reliably attracted more flies than the most attractive media from previous comparisons (5% SC) (Fig. 6 and Table 6).

**Table 6:** Nitrogen composition of media used in this study.

**Media**	**Ammonium Sulfate (per L)**	**Amino Acids (per L)**	**Glucose (per L)**
YNB	5.0g	0	5.0g
SC	0	2.0g	5.0g
YPD	>5.0g*	>2.0g*	5.0g

*YPD is a complete media source made by combining yeast extract and peptone as a nutrient and nitrogen source. The exact nitrogen composition of these products is unknown, but each is used in excess in a typical YPD media recipe (represented above as >5.0g/L and >2.0g/L)

## Discussion

In this work, we have identified a piece of the volatile vocabulary that helps bring together *S. cerevisiae* and *D. melanogaster* in nature. *S. cerevisiae* is a versatile microbe that can survive and propagate on many nutritional sources. Different combinations of substrate and genotype impose unique metabolic demands on the yeast, leading to the production of distinct volatile profiles. These characteristic volatile fingerprints can, in turn, signal to other organisms, such as *D. melanogaster*, information about the substrate, and, potentially, its suitability for their purposes.

Our results show that mitochondria affect *S. cerevisiae* metabolism even during periods of fermentation, and that these metabolic alterations dramatically influence the volatile small molecules produced and released by *S. cerevisiae*. In this way, *S. cerevisiae* cells with and without functional mitochondria, grown on the same substrate, produce different volatile profiles. We have shown that *D. melanogaster* are able to sense the differences in the volatiles produced by these strains and prefer the volatile “fingerprint” produced by yeast strains with mitochondria. Using GC-MS, we identified the molecular compounds produced at higher levels in the attractive cultures, and tagged these compounds as mitochondrially-associated attractants.

We also found that varying the type of nitrogen provided to a culture changes the levels and varieties of volatiles it produces, including our mitochondrially-associated attractants. This makes sense, as metabolism of many nitrogen sources are known to be associated with mitochondria. We found the highest levels of our attractants when the wild-caught *S. cerevisiae* strain was grown on a media substrate with a nutritional composition similar to that of fruit. Like fruit, the media contained high levels of fermentable sugars and a mixture of amino acids as a nitrogen source—a nutritional scenario lending towards a combination of fermentation and mitochondrial activity.

Mitochondrial presence and nitrogen source influence the production of attractants during fermentation. This suggests that amino acid metabolism during fermentation determines volatile compound production. In addition, it is known that the attractive ethyl esters are formed from fermentation byproducts, ethanol and aliphatic acids, largely via EEB1 activity in an ATP-dependent manner. It may be that mitochondria-linked ATP production even during periods of fermentation is required to efficiently generate these compounds.

In either case, it is unlikely that *D. melanogaster* have evolved to recognize the presence of yeast with a functioning mitochondria. Rather we believe our observation of the limited attractiveness of flies to yeast lacking mitochondria has revealed the influence of mitochondria on volatile compound production during a phase of yeast growth—active fermentation—when food for larvae (yeast cells) is abundant and will likely continue to be for some time and when the ongoing production of ethanol by yeast cells created an ideal environment for *D. melanogaster* larvae to develop.

## Methods

### Behavioral setup

A custom-crafted behavior arena and assay protocol was developed for these experiments. Five behavior assays were conducted in parallel. Twenty-two hours (4pm) prior to starting a trial, the specific yeast strains to be tested were streaked from stock plates (stored at 4C and re-streaked from freezer stock (−80C) every two weeks) onto freshly prepared 60×15mm Petri dish plates (see ‘Media Preparation’ for details). Plates were then transferred to 30C incubator and grown overnight. Twenty-two hours later (2pm, following day) plates were removed from incubator and behavioral traps were assembled for the assay.

To construct the traps, the cultures were topped with a custom-designed and 3D printed lid (uPrint Plus), which was then secured into place with Parafilm. The custom lid allowed for the attachment of a 50 mL Falcon tube (bottom sawed off to create a long, open tube) above the plate. A small piece of metal mesh was secured between the adapter and falcon tube to allow for the volatiles emitted from the culture to dissipate up through the Falcon tube, while physically separating the plate from the Falcon tube chamber. The Falcon tube chamber was finally topped with a funnel fashioned out of Whatman Grade 1 Filter paper (15.0cm diameter). The funnel was secured in place by a small piece of lab tape (S5 Fig.).

The constructed traps were next transferred into the behavior chambers: 24” high, 12” diameter clear, open-ended, acrylic cylinders (TAP plastics) fitted with closable sleeve that allowed for placement and removal of both the traps and flies (S5 Fig.). Behavior chambers were kept in a large room with over-head, diffuse lighting that remained on for the entirety of the trial. The chambers were vacuumed and cleaned with water and ethanol before each trail. Four traps were placed in each cleaned arena, two for each yeast strain under analysis. Each strain pair was tested in three different orientations: 2×2 (with the same strain next to each other) in each orientation and a diamond orientation (with the same stain across from the other). One hundred and twenty 4–10 day old *D. melanogaster* were added into the behavioral chamber between 3 and 3:30 pm (see “Fly Husbandry and Handling” section for details). The traps were removed from the chambers 18h later, between 10 and 10:30 am. Before removal the orientation of each trap in each arena was recorded. Flies in each trap were then sexed, counted, and recorded. These raw counts were used to determine the preference for each strain in each arena. The raw data can be found in S1 Data.

### Calculation of Fly Preference (Preference Index)

To quantify and compare independent behavioral trials, we used a simple preference index:

Every behavioral trial contained four traps. Two baited with yeast strain “A” and two baited with strain “B”. One hundred and twenty 4–10 day old *D. melanogaster* were added into each behavioral trial:

A = Total *D. melanogaster* caught in both traps baited yeast strain “A” in the trial
B = Total *D. melanogaster* caught in both traps baited yeast strain “B” in the trial

Preference Index(PI)=A−B/(A+B)/2

### Fly Husbandry and Handling

*D. melanogaster* line Raleigh 437 was obtained from Bloomington Stock Center for use in these experiments. All *D. melanogaster* used in behavioral assays were raised using the same regimen. A “time course” for each line was maintained for the duration of the experiments. The time course consisted of pushing 3 vials of 25 flies into fresh media (CSM formula) topped with dried yeast pellets (Red Star) daily. The 25 flies were refreshed with 4-day-old flies from a stock population every 10 days. The time course of vials was kept at 18C and the eggs laid in each vial were allowed to develop (~18 day generation time). After ~18 days, each seeded vial was checked daily for newly eclosed flies. All flies hatching off of on a given day were transferred into fresh vials and stored in a 25C incubator for at least 4 days to ensure full development and maturation to adulthood. The entire process was repeated daily to maintain each strain.

To prepare flies for a behavioral trial, aged 4–10 day old flies were removed from the 25C incubator and anaesthetized using CO2 and counted into groups of 120. The groups were visually inspected to insure roughly equal numbers of males and females. The flies were transferred into a vial of fresh food and allowed to recover and eat for 2–4 hours before being added into the behavior chamber.

A stock of Raleigh 437 was maintained at 18C and transferred to fresh media monthly.

### Media Preparation

5% YPD for behavioral assays and strain storage was prepared weekly using the following recipe (for 1L): 20g Peptone (BD Bacto Peptone), 10g Yeast Extract (Amresco Yeast Extract, Bacteriological, Ultra Pure Grade), 50g Dextrose (Fisher Scientific Dextrose Anhydrous), 20g Agar (BD Bacto Agar), Water to 1L. Solution was mixed and heated to boiling on a magnetic stir plate. Liquid YPD was prepared using (for 1L): 20g Peptone, 10g Yeast Extract, 50g Dextrose, Water to 1L. The solution was stirred for five minutes and filter sterilized using Nalgene vacuum filtration system (0.22 micron).

The following additional recipes were used in this experiment

5% SC (for 1L). 1.7g YNB without amino acids and ammonium sulfate (BD Difco), 2.0g SC Amino acid mixture (MP Biomedicals), 50g Dextrose, 20g Agar, Water to 1L.

5%YNB (for 1L). 6.7g YNB without amino acids (BD Difco), 50g Dextrose, 20g Agar, Water to 1L.

Grape Agar (for 1L). 1.7g YNB without amino acids and ammonium sulfate, 1L organic grapes (v/v) (thoroughly washed and pureed in food processor), 20g Agar, Water to 1L.

### Yeast strains

Strains used in this study are listed in Table 7 and Table 8.

**Table 7:** Yeast strains used in this study.

**Strain**	**Genotype**	**Source**
BY4741	*MATa;his3;leu2;met15;ura3*	ATCC
BY4742p	*MATα;his3;leu2;lys2;ura3*	This Study
BY4742g	*MATα;his3;leu2;lys2;ura3*	ATCC
MRPL16Δ (BY4742)	*MATα;his3;leu2;lys2;ura3;mrpl16*	Calahan et al (2011)[43]
EEB1Δ (BY4741)	*MATa;his3;leu2;met15;ura3;eeb1*	ATCC
T73 (ATCC28383)	Wild isolate	Hyma and Faye(2013)[44]

**Table 8:** Yeast strains used in the tetrad experiment.

**Strain**	**Genotype**	**Source**
1A (YME01)	MATα;met15;lys2;his3;ura3;leu2	This study
1B (YME02)	MATα;met15;LYS2;his3;ura3;leu2	This study
1C (YME03)	MATa;MET15;LYS2;his3;ura3;leu2	This study
1D (YME04)	MATa;MET15;lys2;his3;ura3;leu2	This study
3A (YME05)	MATα;MET15;lys2;his3;ura3;leu2	This study
3B (YME06)	MATa;met15;LYS2;his3;ura3;leu2	This study
3C (YME07)	MATα;MET15;lys2;his3;ura3;leu2	This study
3D (YME08)	MATa;met15;lys2;his3;ura3;leu2	This study
4A (YME09)	MATa;MET15;LYS2;his3;ura3;leu2	This study
4B (YME10)	MATα;met15;lys2;his3;ura3;leu2	This study
4C (YME11)	MATa;met15;LYS2;his3;ura3;leu2	This study
4D (YME12)	MATα;MET15;lys2;his3;ura3;leu2	This study
10A (YME13)	MATa;met15;lys2;his3;ura3;leu2	This study
10B (YME14)	MATα;MET15;LYS2;his3;ura3;leu2	This study
10C (YME15)	MATa;MET15;lys2;his3;ura3;leu2	This study
10D (YME16)	MATα;met15;LYS2;his3;ura3;leu2	This study
18A (YME17)	MATa;met15;lys2;his3;ura3;leu2	This study
18B (YME18)	MATα;met15;lys2;his3;ura3;leu2	This study
18C (YME19)	MATa;MET15;LYS2;his3;ura3;leu2	This study
18D (YME20)	MATα;MET15;LYS2;his3;ura3;leu2	This study

### Tetrad Experiment

Twenty tetrads were obtained using a standard sporulation protocol (Methods in Yeast Genetics, 2005). Briefly, BY4741 and BY4742p were mixed on a YPD agar plate to encourage mating. Diploid colonies were selected by replica plating onto a selective media (Met(-);Lys(-)) transferred to liquid “YPA” media for 1 day, spun down and resuspended in “Minimal Spo” for at least 3 days. Ascii were dissolved and tetrads dissected. After incubation, tetrads were genotyped by replica plating onto selective media for MAT, LYS, and MET loci. Genotyped tetrads were grown in YPD and prepared as freezer stocks using standard protocol (Methods in Yeast Genetics, 2005). Freezer stocks of each stain were used to start cultures for all behavior experiments.

Strains used in the tetrad experiment are listed in Table 8.

### GC-MS protocol

Sample analysis was performed on Agilent 7890A/5975C GC-MS equipped with a HP-5MS (30m × 0.25mm, i.d., 0.25micrometers film thickness) column.

To sample the headspace of a plate culture, a conditioned, Twister stir bar (10 mm in length, 0.5mm film thickness, 24microliters polydimethylsiloxane volume) was suspended from the lid of the Petri dish using rare earth magnets for 40 minutes at room temperature. The Twister was then dried using a Kimwipe, placed in a Gerstel thermal desorption sample tube, topped with a transport adapter, and loaded into sampling tray.

Automated sampling and analysis was performed using the Gerstel MPS system and MAESTRO integrated into Chemstation software.

Samples were thermally desorbed using the Gerstel Thermal Desorption Unit (TDU), followed by injection into the column with a Gerstel Cooled Injection System (CIS-4). Temperature program for desorption was 30C (.40 min), then 60C/min to 250C(hold for 5 mins). Temperature of the transfer line was set at 275C. The injection was cooled with liquid nitrogen to −100C with a hold time (.60min). The injector inlet was operated in the Solvent Vent mode, with a vent pressure of 9.1473 psi, a vent flow of 30mls/min, and a purge flow of 6mls/min (for .01 min).

The following GC-MS method parameters was used for each sample: The GC temperature program was 40C (2 mins), then 4C/min to 140C (hold 0 mins), then 15C/min to 195C (hold for 0 min). The carrier gas head pressure was 9.1473 psi for a flow rate of 1.2mL/min. The GC was operated in the constant flow mode. The MS was operated in EI mode with the electron voltage set at autotune values. The detector was set to scan from 30 to 300amu at a threshold of 150 with 3 samples for a scanning rate of 2.69 scans/second. The MSD transfer line temperature was set at 280C. The ion source and quadrupole temperatures were set at 230C and 150C, respectively.

GC-MS data files were visually inspected using Chemstation. TIC peaks were identified using the NIST O8 database via Chemstation. Datafiles were transferred, parsed, and analyzed using custom written Matlab scripts (see S1 Scripts). Every chromatogram trace represents at minimum, the average of 3 biological replicates.

To verify the compound calls made through the NIST 08 database, we prepared 1:1000 (in paraffin oil) dilutions of purchased synthetic standards (Sigma). The dilations were sampled, run, and processed using the same parameters as the fermentations. The chromatograms were overlaid with a BY4741 chromatogram final confirmation (S6 Fig.).

### Aerobic and anaerobic yeast cultures

Pairs of 100mL 5% YPD cultures were inoculated with BY4741 or BY4742p cells (stored on a solid 5% YPD plate at 4C) and topped with either a loose foil cap or fermentation bung (Ferm-Rite) to create aerobic and anaerobic growth conditions for each strain. Cultures were grown for 21h shaking at room temperature. After this growth period, cultures were removed from shaker, and an O.D. reading was taken to ensure similar cell densities across each aerobic and anaerobic culture pair. 50mL of each culture was transferred to a .22 micron vacuum filter (MilliQ Steri-Flip) to remove yeast from the culture medium. 20mL of the culture medium was then transferred into 20mL amber glass vials (Sigma), a conditioned Twister stir bar (Gerstel) was added, and the vial was capped with a silica-lined screw cap (Sigma). The Twister stir bars were spun in the medium at 300rpm for 40min, rinsed with MilliQ water, dried with a Kimwipe, and transferred onto the GC-MS for analysis (see “GC-MS protocol” for details).

### Glycerol plate growth

BY4741 and BY472p and tetrads representing each genotype were streaked from 5% YPD stock plates onto 5% glycerol plates. Plates were incubated at 30C for 48h and visually inspected for growth.

### Sequencing

*S. cerevisiae* DNA was extracted using the Qiagen Gentra Purgene Kit and sheared using the Diagenode Bioruptor Standard. Sheared DNA was checked for quality using Qubit dsDNA HS Assay and an Agilent BioAnalyzer. DNA libraries (350 base pair insert size) were prepared using an Illumina TruSeq DNA PCR-Free Sample Preparation Kit and were sequenced on an Illumina HiSeq 2000 Platfrom at the Vincent J. Coates Genomics Sequencing Laboratory (UC Berkeley). Seqeunce reads were mapped back to the *S.cerevisiae* reference genome (SGD) using Bowtie.

## Supporting Information

S1 Data Raw data from behavior experiments.
(XLSX)

S1 Fig Tetrad produces GC-MS profile similar to BY4741 at identified attractants. Averaged TICs for BY4741, BY4742p and a selected tetrad (*MATα;MET15;lys2*). BY4741 and BY4742p are plotted on the positive y-axis and the tetrad on the negative y-axis in each panel. TICs in each panel were normalized together so that the maximum value across both experiments was set to one.
(TIF)

S2 Fig Comparison of GC-MS profiles of aerobically and anaerobically grown BY4741 and BY4742p cultures. Averaged TICs for: A) BY4741 grown both aerobically and anaerobically and B) BY4742p grown aerobically and anaerobically in 5% YPD. TICs in each panel were normalized together so that the maximum value across both experiments was set to one. In each comparison, aerobic cultures are plotted on the positive y-axis and the anaerobic cultures plotted on the negative y-axis.
(TIF)

S3 Fig Deletion of EEB1 decreases ethyl ester production and affects *D. melanogaster* preference. Results of preference assay comparing BY4741 and BY4741 ΔEEB1 (left), along with averaged TICs for GC-MS analysis of for BY4741 (positive y-axis) and BY4741 ΔEEB1 (negative y-axis). Both TICs were normalized together so that the maximum value across both experiments was set to 1.
(TIF)

S4 Fig GC-MS comparison of various wild-caught *S.cerevisiae* isolates grown on YPD and YNB. Multiple wild isolates (YPS 163, T73, Clib 382, I14, and RM 11) were grown on YPD and YNB, two media with equivalent amounts of the sugar (5% glucose) and varying types of nitrogen (Table 6). Average TIC chromatograms were determined for each media—strain combination, as well as the area under the peak for each of the primary and secondary attractants. Next, the amount of each attractant was compared for the two media types (YPD and YNB) for each wild isolate. The relative difference between the levels was plotted on the positive axis if larger in YPD or on the negative axis if the peak was larger in YNB. The relative amounts of all identified attractants track similarly across all six strains tested.
(AI) Click here for additional data file.

S5 Fig Images of the behavioral trap (A) and behavioral arena (B) explained in “Behavioral setup” section of Methods.
(TIF)

S6 Fig GC-MS of Synthetic Standards verify the identity of primary attractants. GC-MS chromatograms of synthetic standards representing the primary attractants identified in this study overlaid with a BY4741 TIC. The synthetic standards match with the peaks representing primary attractants in the BY4741 TIC further verifying the identity of these compounds.
(TIF)

S1 Scripts Scripts used in analysis of GC-MS data.
(ZIP)

S1 Table Proportion of sequence reads that map to the mitochondrial genome.
(DOCX)
