# Supplementary figures and images for: Saccharomyces cerevisiae mitochondria are required for optimal attractiveness to Drosophila melanogaster
Source: PLoS One. 2014 Dec 2;9(12):e113899. doi: 10.1371/journal.pone.0113899 (PMC4252075; doi:10.1371/journal.pone.0113899)

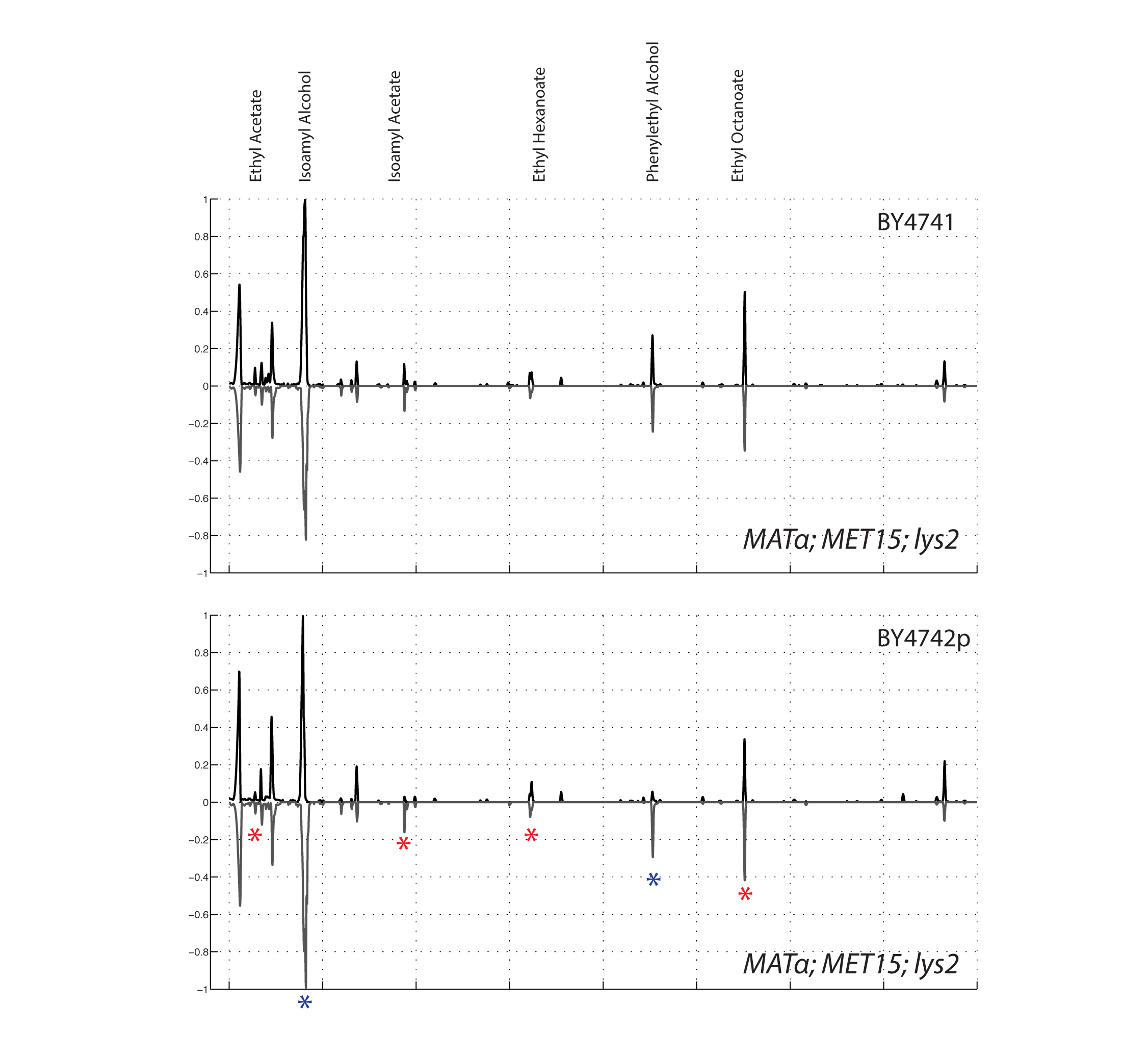

Supplement: S1 Fig — Averaged TICs for BY4741, BY4742p and a selected tetrad (MATα;MET15;lys2). BY4741 and BY4742p are plotted on the positive y-axis and the tetrad on the negative y-axis in each panel. TICs in each panel were normalized together so that the maximum value across both experiments was set to one. (TIF) [file pone.0113899.s002.tif]

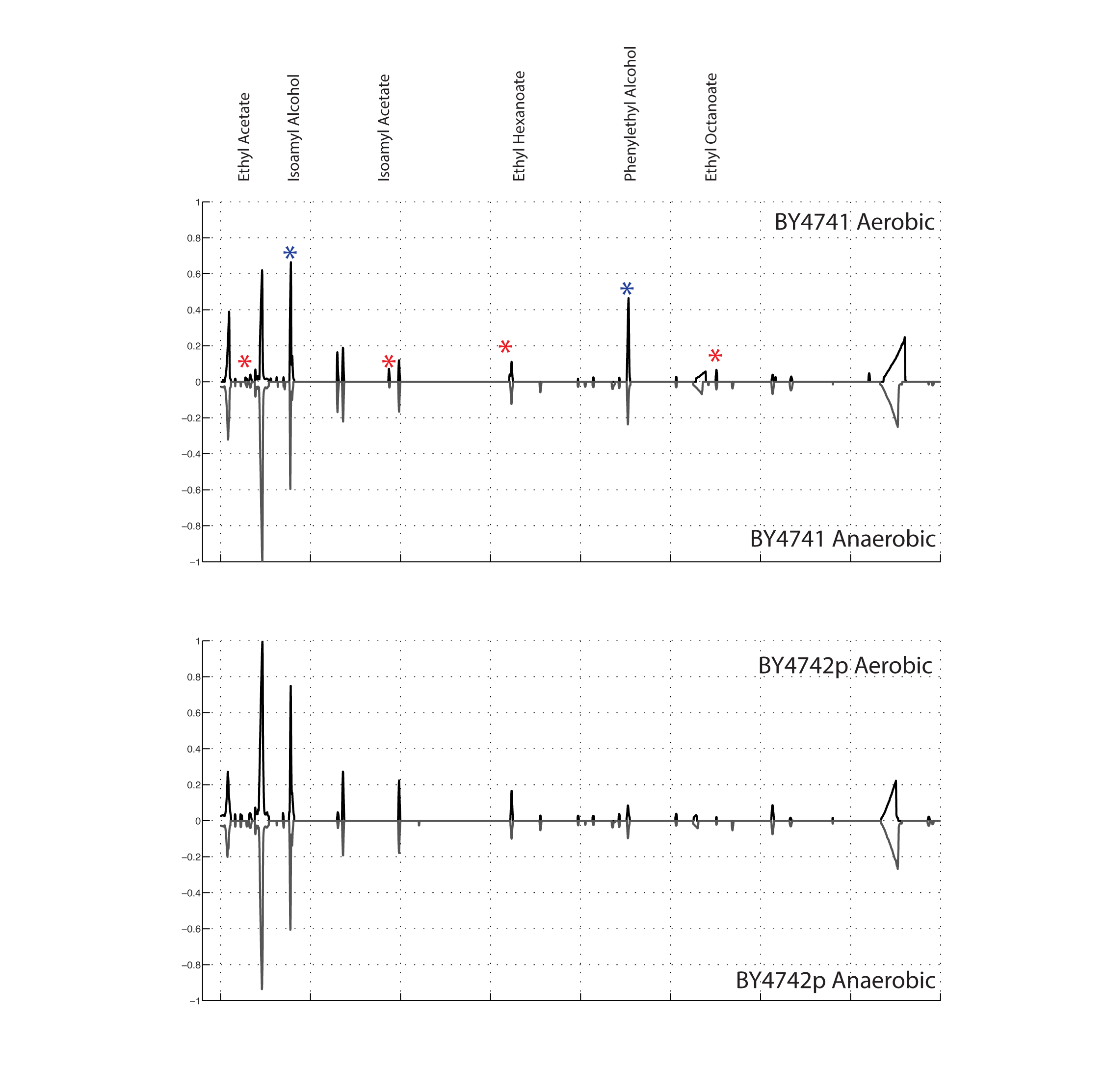

Supplement: S2 Fig — Averaged TICs for: A) BY4741 grown both aerobically and anaerobically and B) BY4742p grown aerobically and anaerobically in 5% YPD. TICs in each panel were normalized together so that the maximum value across both experiments was set to one. In each comparison, aerobic cultures are plotted on the positive y-axis and the anaerobic cultures plotted on the negative y-axis. (TIF) [file pone.0113899.s003.tif]

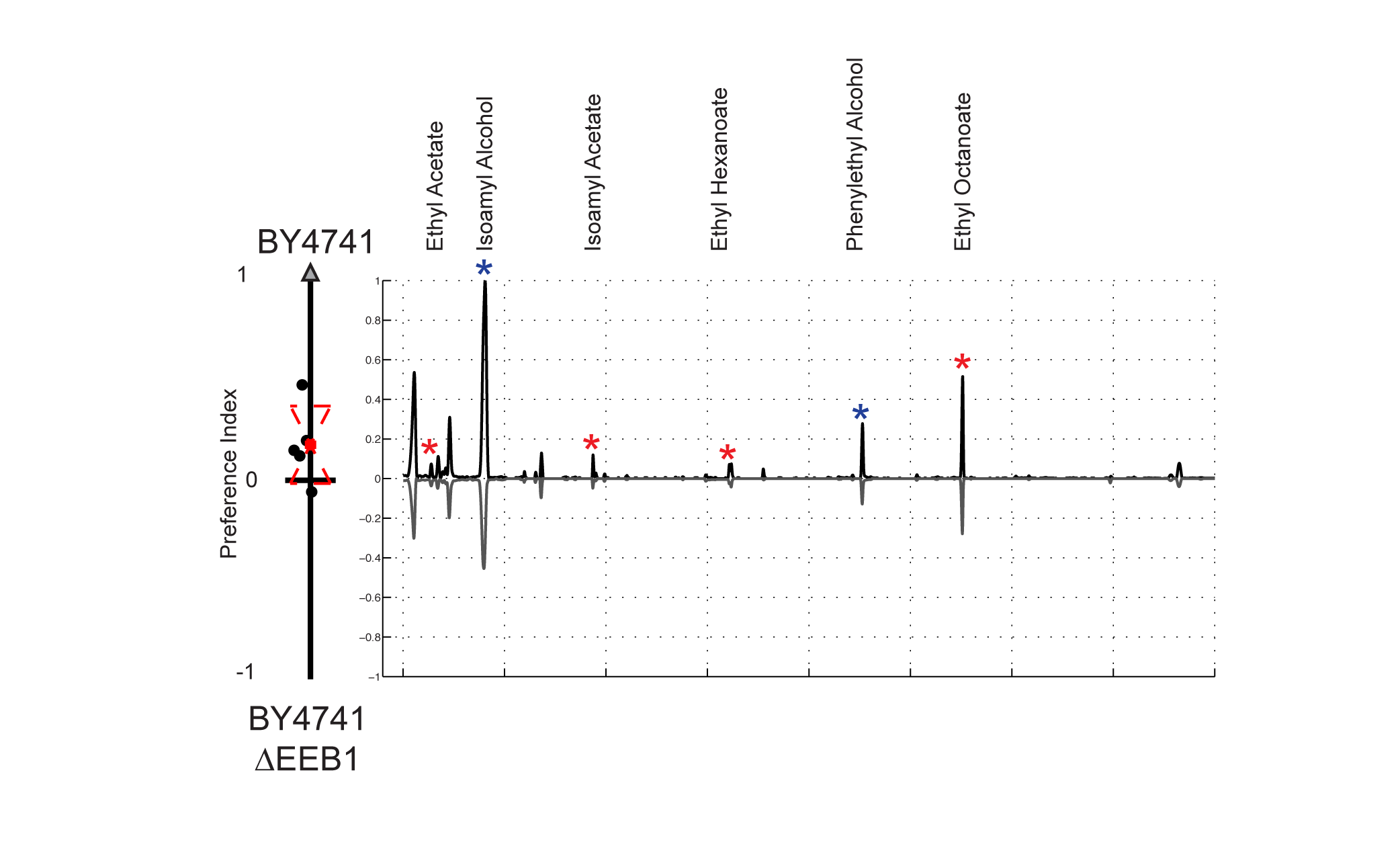

Supplement: S3 Fig — Results of preference assay comparing BY4741 and BY4741 ΔEEB1 (left), along with averaged TICs for GC-MS analysis of for BY4741 (positive y-axis) and BY4741 ΔEEB1 (negative y-axis). Both TICs were normalized together so that the maximum value across both experiments was set to 1. (TIF) [file pone.0113899.s004.tif]

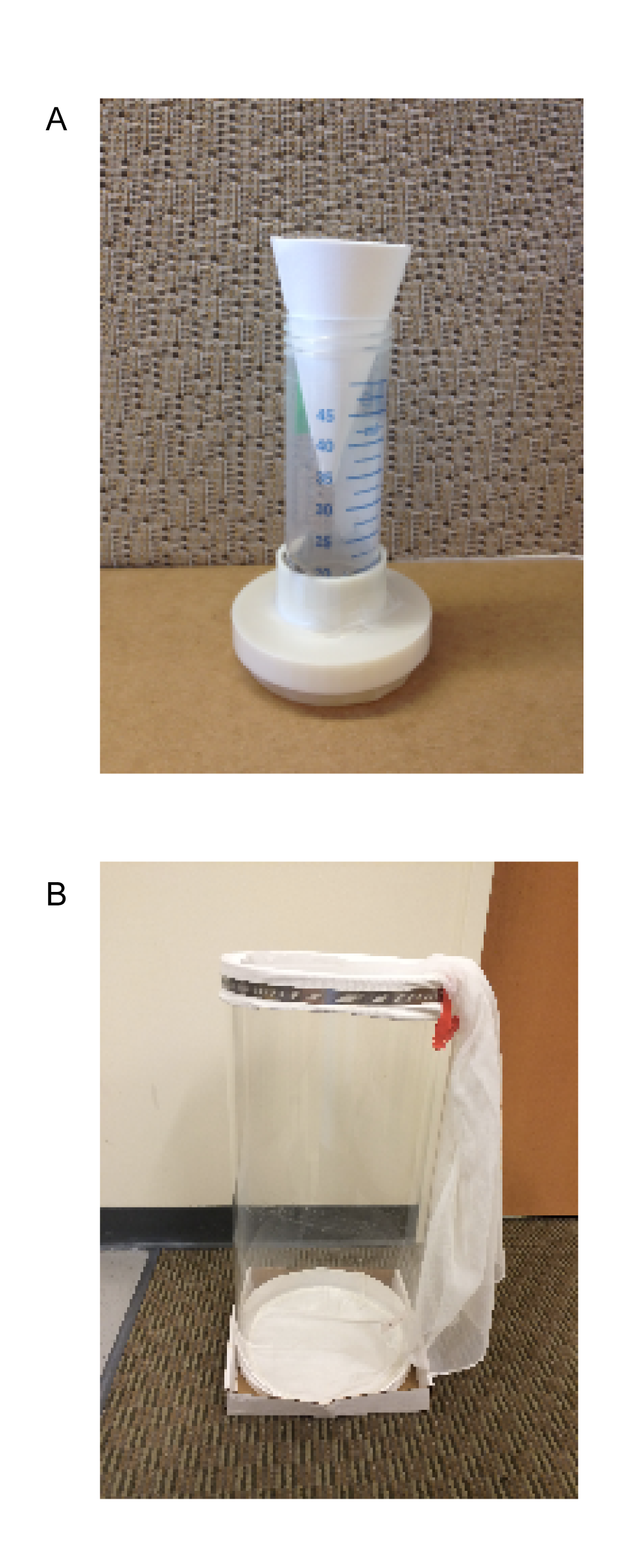

Supplement: S5 Fig — Images of the behavioral trap (A) and behavioral arena (B) explained in “Behavioral setup” section of Methods. (TIF) [file pone.0113899.s006.tif]

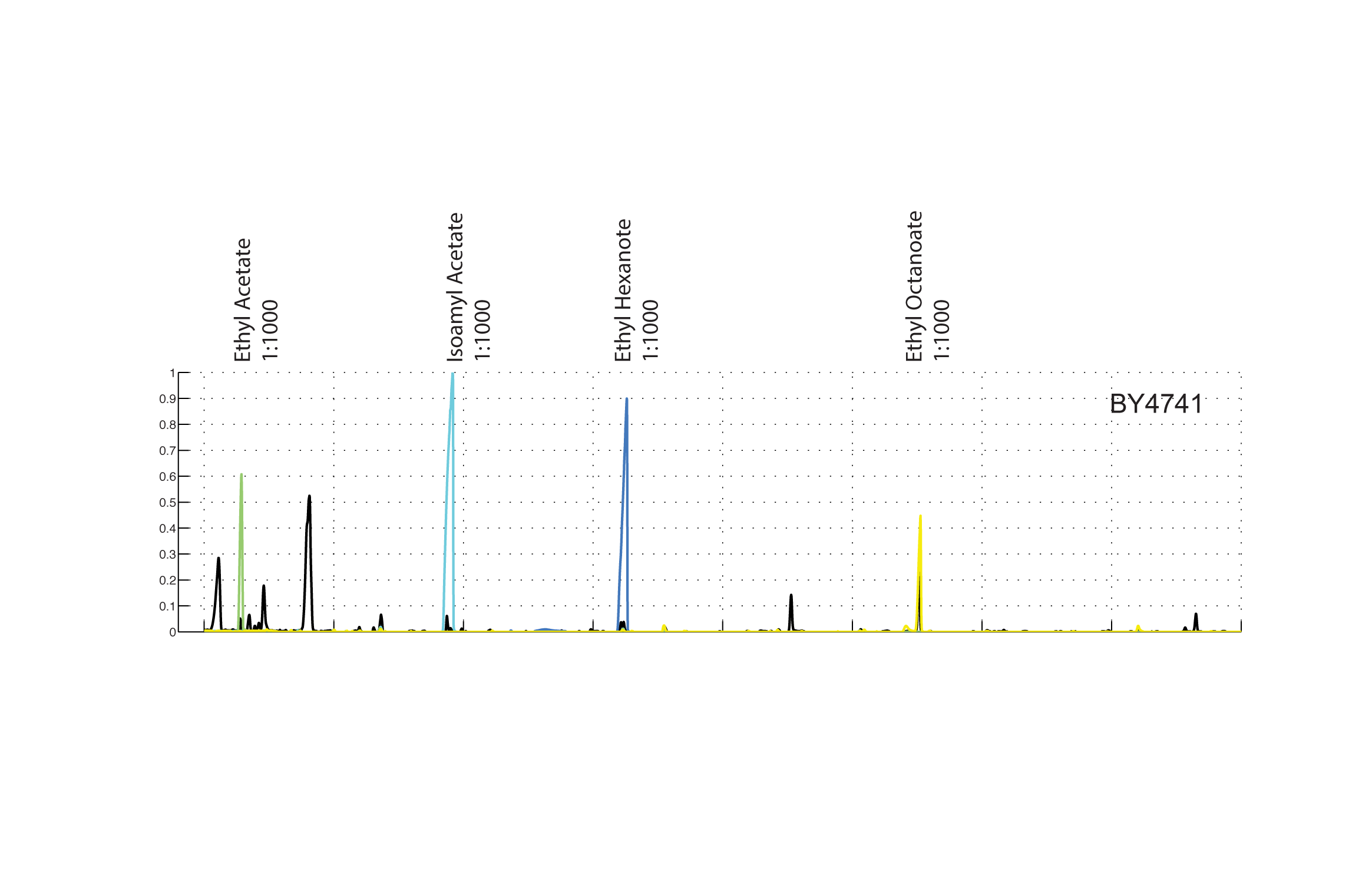

Supplement: S6 Fig — GC-MS chromatograms of synthetic standards representing the primary attractants identified in this study overlaid with a BY4741 TIC. The synthetic standards match with the peaks representing primary attractants in the BY4741 TIC further verifying the identity of these compounds. (TIF) [file pone.0113899.s007.tif]
