# Supplementary material for: Saccharomyces cerevisiae mitochondria are required for optimal attractiveness to Drosophila melanogaster
Source: PLoS One. 2014 Dec 2;9(12):e113899. doi: 10.1371/journal.pone.0113899 (PMC4252075; doi:10.1371/journal.pone.0113899)
Supplement: S1 Table — Proportion of sequence reads that map to the mitochondrial genome. (DOCX) [file pone.0113899.s009.docx]

Supplemental Table 1: Proportion of sequence reads that map to the mitochondrial genome

| **Strain** | **Mitochondrial Reads** | **Total Reads** | **Percentage** |
| --- | --- | --- | --- |
| BY4741 | 247769 | 40808998 | 0.6070% |
| BY4742p | 929 | 23844974 | 0.0039% |
| BY4742g | 148834 | 24066666 | 0.6180% |
